# Soliton Fractional Charges in Graphene Nanoribbon and Polyacetylene: Similarities and Differences

**DOI:** 10.3390/nano9060885

**Published:** 2019-06-14

**Authors:** S.-R. Eric Yang

**Affiliations:** Department of Physics, Korea University, Anam-dong 5, Seongbuk-gu, Seoul 02481, Korea; eyang812@gmail.com

**Keywords:** chiral symmetry, fractional charge, topological insulator, soliton, graphene nanoribbon

## Abstract

An introductory overview of current research developments regarding solitons and fractional boundary charges in graphene nanoribbons is presented. Graphene nanoribbons and polyacetylene have chiral symmetry and share numerous similar properties, e.g., the bulk-edge correspondence between the Zak phase and the existence of edge states, along with the presence of chiral boundary states, which are important for charge fractionalization. In polyacetylene, a fermion mass potential in the Dirac equation produces an excitation gap, and a twist in this scalar potential produces a zero-energy chiral soliton. Similarly, in a gapful armchair graphene nanoribbon, a distortion in the chiral gauge field can produce soliton states. In polyacetylene, a soliton is bound to a domain wall connecting two different dimerized phases. In graphene nanoribbons, a domain-wall soliton connects two topological zigzag edges with different chiralities. However, such a soliton does not display spin-charge separation. The existence of a soliton in finite-length polyacetylene can induce formation of fractional charges on the opposite ends. In contrast, for gapful graphene nanoribbons, the antiferromagnetic coupling between the opposite zigzag edges induces integer boundary charges. The presence of disorder in graphene nanoribbons partly mitigates antiferromagnetic coupling effect. Hence, the average edge charge of gap states with energies within a small interval is e/2, with significant charge fluctuations. However, midgap states exhibit a well-defined charge fractionalization between the opposite zigzag edges in the weak-disorder regime. Numerous occupied soliton states in a disorder-free and doped zigzag graphene nanoribbon form a solitonic phase.

## 1. Introduction

Graphene has considerable potential, not only for spintronic applications, but also for fundamental physics [1,2]. In particular, graphene systems have topologically protected chiral zigzag edge modes [3,4]. An excellent opportunity to observe these boundary charges has recently arisen, as rapid progress has been made in the fabrication of atomically precise graphene nanoribbons (GNRs) [5,6,7]. Thus, the generation mechanism of solitons/vortices is at the center of research on graphene materials [8].

**Figure 1:**
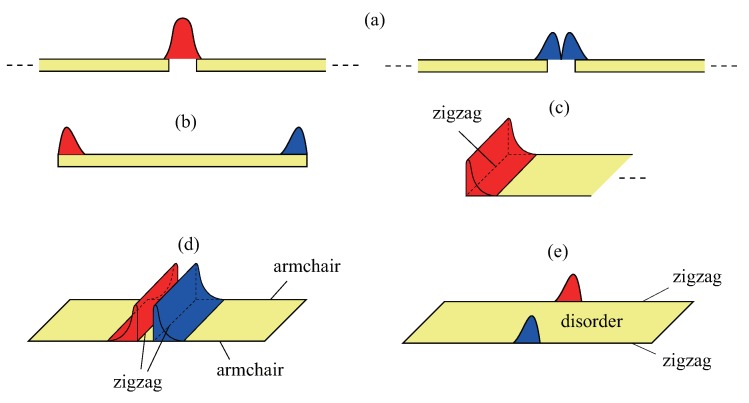
Possible soliton states discussed in this paper. The probability densities of a soliton on A and B carbon atoms are indicated by red and blue colors, respectively. (**a**) Chiral zero modes of polyacetylene: the domain-wall soliton (left) and antisoliton (right) have different chiralties. (**b**) End-state soliton of polyacetylene with charge fractionalization: this soliton can be represented by the bonding or antibonding linear combination of the chiral end states. (**c**) Chiral zero mode on zigzag edge of semi-infinte armchair graphene ribbon. (**d**) Domain-wall soliton of armchair graphene nanoribbon under local tensile strain. The probability densities on the A and B carbon atoms have a small overlap. (**e**) Charge fractionalization on zigzag edge of interacting disordered zigzag nanoribbon. The probability densities on the A and B carbon atoms are well separated.

An object of one-dimensional insulators qualifies as a soliton if it derives half its fractional spectral weight from each of the conduction and valence bands. Jackiw and Rebbi [9] showed that a twist in the scalar mass potential in the Dirac equation, connecting two degenerate groundstates, can produce a zero-energy soliton state. Furthermore, the independent work of Su, Schrieffer, and Heeger [10,11] showed the presence of a soliton (kink) in polyacetylene. This kink exists between two different dimerized phases and is called a domain-wall soliton (see Figure 1a). A domain wall supports either a soliton or an antisoliton, but not both. A many-body ground state with the solitonic state unoccupied by an electron has an unusual charge *Q* and spin *S* relation: Q=e and S=0 (here, the contribution from the positive ion background charge is included; e>0 is the elementary charge). When the solitonic state is occupied, the following values are obtained: Q=0 and S=1/2. In finite-length polyacetylene, a soliton may exist with fractional *boundary* charges at the two end points (one for each end), as shown in Figure 1b.

A domain-wall soliton can also exist in a GNR [12,13,14] (see Figure 1d). A single domain wall in a GNR can support both a soliton and antisoliton, in contrast to polyacetylene; consequently, no unusual spin and charge relation holds. In a doped zigzag GNR (ZGNR), numerous soliton states can be occupied, forming a solitonic phase [15]. However, no fractional *boundary* charges are found in a GNR. This is due to *antiferromagnetic* coupling between the zigzag edges [3]. In the presence of disorder, undoped GNRs with zigzag edges form a Mott-Anderson insulator, and the effect of antiferromagnetic coupling between the opposite zigzag edges is partly mitigated. Hence, in the weak–disorder regime, a midgap state can have e/2 fractional boundary charges on the opposite zigzag edges, i.e., one for each edge [16], as shown in Figure 1e (zigzag edges are located along the ribbon direction in ZGNRs and not at the end points). Disorder acts as a singular perturbation on zigzag edge states. This is a consequence of a non-trivial interplay between disorder and electron interactions. Note that an interacting disordered ZGNR has also non-trivial localization properties: the gap states are localized whereas the states outside the gap are delocalized, and the usual localization theory does not apply.

In this work, the similarities and differences between solitons in GNRs and polyacetylene are explained in detail. Various solitons of GNRs and polyacetylene are shown schematically in Figure 1. One important common feature is the chiral symmetry, which guarantees that topological edge states exist in both systems. This feature is manifested by the bulk-edge correspondence between the Zak phase and the existence of edge states. The *chiral boundary modes* are shown to be intimately related to the fractional boundary charges, which are formed from mixed chiral modes of bonding or antibonding linear combinations of chiral modes. The main differences between GNRs and polyacetylene are “fermion doubling” and the role of electron-electron interactions in charge fractionalization, as explained below. It is hoped that this introductory overview of solitons will improve understanding of fractional boundary charges and stimulate experimental searches for e/2 charges in GNRs. Detection of these fractional charges may facilitate the study of particles that obey fractional statistics in ZGNRs.

This paper is organized as follows. In Section 2, the connection between the boundary modes and the Zak phase (bulk-edge correspondence), which is based on chiral symmetry, is explained. In Section 3, a short review of the formation of domain-wall and end solitons with fractional end charges in polyacetylene is provided. Domain-wall and end solitons of GNRs are reviewed in Section 4. The formation of fractional boundary charges in ZGNRs is more complex than that in polyacetylene, because of the antiferromagnetic coupling between the well-separated zigzag edges. In Section 5, we argue that disorder partially mitigates the effect of the edge antiferromagnetism and induces formation of fractional charges of the midgap states. The conclusion is presented in Section 6.

## 2. Chiral Symmetry, Zak Phase, and Boundary Modes

Before describing the topological properties of solitons in polyacetylene and GNRs, the importance of the chiral symmetry of these systems is explained. The presence of topological boundary states in GNRs and polyacetylene is intimately related to the presence of chiral symmetry (sublattice symmetry) [4]. This behavior yields *bulk edge correspondence*, for which an edge mode must exist when the bulk topological Zak phase is π.

### 2.1. Chiral Symmetry

Polyacetylene and GNRs have two inequivalent sublattices (here labeled A and B). Under the chiral operation Γ, the site annihilation operators transform according to $a_{iA}\to a_{iA}$ and $a_{iB}\to -a_{iB}$, where the index *i* denotes a unit cell containing one A and one B carbon atom. The nearest-neighbor tight-binding Hamiltonians of polyacetylene and GNRs satisfy the anticommutation relation {Γ,H}=0. This symmetry is not exact when next nearest-neighbor hopping is included. However, it yields a massless Dirac equation, which well describes low-energy excitations. Single-particle eigenstates with non-zero eigenenergies *E* and −E are related by the chiral operation $\Gamma |\psi_{E}\rangle =|\psi_{-E}\rangle$. Thus, chiral symmetry implies particle-hole symmetry. The probability densities of $|\psi_{E}\rangle$ and $\Gamma |\psi_{E}\rangle$ are identical. Of special interest are the single-particle eigenstates of the chiral operator, which represent chiral topological states.

### 2.2. Zak Phase and Edge Charge

One can show that the presence of a boundary charge in a system with chiral symmetry can be related to a bulk topological number (see the next subsection). This number is the Zak phase. In this subsection the Zak phase and its relation to the edge charge are discussed. The Zak phase of a one-dimensional band structure is a topological Berry phase acquired by an electron as it moves adiabatically through the first Brillouin zone [17]

(1) $$\begin{array}{c} Z_{1D}=\sum_{l\in occ}i\oint_{B.Z.}\langle u_{lk}|\nabla_{k}u_{lk}\rangle dk, \end{array}$$

where $|u_{lk}\rangle$ is the periodic part of the Bloch wave function of the *l*th band and the sum is over the occupied bands. Note that $Z_{1D}$ is a *bulk* property determined by the band structure of a periodic system. Even when the chiral symmetry is broken, a one-dimensional periodic insulator with inversion/reflection symmetry has $Z_{1D}$ equal to either 0 or π mod 2π [17] (the modular 2π is a consequence of the gauge invariance).

According to the modern theory of polarization, $Z_{1D}$ is related to the bulk polarization of a periodic system [18,19], such that

(2) $$\begin{array}{c} P=\frac{e}{2\pi }Z_{1D}. \end{array}$$

Note that, for one-dimensional polarization, *P* is defined as the dipole moment per length. Consider an *insulating* edge of the finite length system generated by cutting the periodic system. The Zak phase of the periodic system can be related to the magnitude of the edge charge *Q* [19], such that

(3) $$\begin{array}{c} Q=\vec{P}\cdot \hat{n}\;. \end{array}$$

This is an example of a bulk edge correspondence. Note that *Q* is located at the edge and the direction of $\hat{n}$ is perpendicular to the edge. A *rectangular* armchair GNR (AGNR) has two long armchair edges and two short zigzag edges. The possible values of the zigzag edge charge may be computed using the $Z_{1D}$ of the periodic AGNR: $Z_{1D}=2\pi N$, where *N* is an integer [20]. According to (2) and (3), the edge charge is an *integer* with Q=eN. Note that one cannot compute the possible charge values on the zigzag edges of a ZGNR using Equation (3), as these edges are parallel to the ribbon direction. As $Z_{1D}$ is multi-valued Equation (3) gives possible values of *Q* only. The actual value of *Q* must be computed with consideration of the coupling between the edge and bulk. The computed values for polyacetylene and GNRs are given in subsections III B and IV B, respectively.

### 2.3. Zak Phase of Polyacetylene

The chiral symmetry of polyacetylene yields a non-trivial topological Zak phase [4]. It is instructive to explicitly compute the Zak phase of polyacetylene and to relate it to a fractional end charge. Consider an infinitely long polyacetylene specimen. The tight-binding Hamiltonian is

(4) $$\begin{array}{c} H=-t\vec{g}\left(k\right)\cdot \vec{\sigma }, \end{array}$$

and the chiral operator is $\Gamma =\sigma_{z}$. Chiral symmetry means $\{\sigma_{z},H\}=0$. This anticommutation relation implies that $\sigma_{z}$ is absent from *H*. This point is crucial as it implies that the vector $\vec{g}\left(k\right)$ does not have a z-component [21]. It can be written as

(5) $$\begin{array}{c} \vec{g}\left(k\right)=\left|\rho \left(k\right)\right|\left(\begin{array}{c} cos\phi (k)\\ sin\phi (k) \end{array}\right), \end{array}$$

where the phase ϕ(k) satisfies $cot\phi \left(k\right)=\frac{t^{\prime }/t}{sinka_{0}}+cotka_{0}$ and $\rho \left(k\right)=t^{\prime }/t+e^{-ika_{0}}$. Here, $t^{\prime }$ and *t* are, respectively, the intra and inter cell hopping parameters. The unit cell length is $a_{0}$. The eigenvectors are

(6) $$\begin{array}{c} u\left(k\right)=\frac{1}{\sqrt{2}}\left(\begin{array}{c} e^{-i\phi (k)}\\ \pm 1 \end{array}\right) \end{array}$$

and the eigenvalues are $\pm t|\vec{g}\left(k\right)|$. The pseudospin of an eigenstate is defined as the expectation value of $\vec{\sigma }$

(7) $$\begin{array}{c} \vec{\Sigma }\left(k\right)=\langle \vec{\sigma }\rangle =\left(\begin{array}{c} cos\phi (k)\\ sin\phi (k) \end{array}\right). \end{array}$$

As *k* varies across the Brillouin zone, the vector $\vec{\Sigma }\left(k\right)$ rotates on a circle. The Zak phase is related to the rotation angle ϕ(k) of $\vec{\Sigma }\left(k\right)$, where

(8) $$\begin{array}{c} Z_{1D}=\frac{1}{2}\oint_{B.Z.}\frac{\partial \phi (k)}{dk}dk. \end{array}$$

As *k* varies across the Brillouin zone, $Z_{1D}$ is given by the difference ϕ(π/a)−ϕ(−π/a). When the unit cell of a periodic polyacetylene specimen contains a long bond ($t^{\prime }/t<1$ ), $Z_{1D}$ is π mod 2π. This reflects the fact that the bulk polarization can depend on the choice of unit cell [18]. In the case of a short-bond unit cell ($t^{\prime }/t>1$ ), $Z_{1D}$ is 0 mod 2π. A topological phase transition exists when the intra- and inter-cell tunneling coefficients are equal, i.e., $t^{\prime }=t$ [21]. In that case, long-bond polyacetylene is topologically non-trivial whereas short-bond polyacetylene is topologically trivial. It can be demonstrated that the non-trivial value of $Z_{1D}$ induces the emergence of edge states in a finite system with open boundary conditions [22]. Such a soliton has an end charge of e/2 (See Figure 1c). This fractional value can also be derived directly from Equations (2) and (3).

### 2.4. Edge Modes of Graphene Sheet and Zak Phase

The bulk-edge correspondence also holds for a semi-inifinte graphene sheet, for which the edge may be a zigzag or armchair. The number of edge modes with wavevector $k_{\|}$ parallel to the edge direction can be determined using the bulk-edge correspondence. For a two-dimensional band structure, one can define an analog of the one-dimensional Zak phase. For a wavevector $k_{\|}$, this Zak phase is defined as

(9) $$\begin{array}{c} Z_{2D}\left(k_{\|}\right)=i\oint_{B.Z.}\langle u_{\vec{k}}|\nabla_{k_{⊥}}u_{\vec{k}}\rangle dk_{⊥}, \end{array}$$

where the wavevector $k_{⊥}$ is perpendicular to $k_{\|}$. Integration over $k_{⊥}$ should be performed on a cut of a 2D Brillouin zone in a direction transverse to $k_{\|}$. One can show that $Z_{2D}\left(k_{\|}\right)/\pi$ gives the number of edge states at $k_{\|}$ (this is an example of the bulk-edge correspondence [4,22]). Then,

(10) $$\begin{array}{c} Z_{2D}\left(k_{\|}\right)=\frac{1}{2}\oint_{B.Z.}\frac{\partial \phi (\vec{k})}{dk_{⊥}}dk_{⊥}. \end{array}$$

When the integration is performed along the direction of an armchair edge, $Z_{2D}\left(k_{\|}\right)$ is of π; however, it is zero when integrated in the direction of a zigzag ribbon. This implies the existence of *chiral* edge states on a zigzag boundary, but not on an armchair edge [4,22]. Tight-binding calculations confirm this result (see subsection IV B). Thus, these edge states are protected topologically. Note that the boundary charge given by Equation (3) cannot be calculated from $Z_{2D}\left(k_{\|}\right)$; one must use $Z_{1D}$ instead. There is no reason why $Z_{1D}$ should be identical to $Z_{2D}\left(k_{\|}\right)$.

## 3. Solitons in Polyacetylene

Topological edge modes are soliton modes. Some salient features of solitons in polyacetylene are briefly reviewed here (see Ref. [11] for a comprehensive review; refs. [21,23] also give a nice overview of solitons).

### 3.1. Domain-Wall Soliton in Polyacetylene

**Figure 2:**
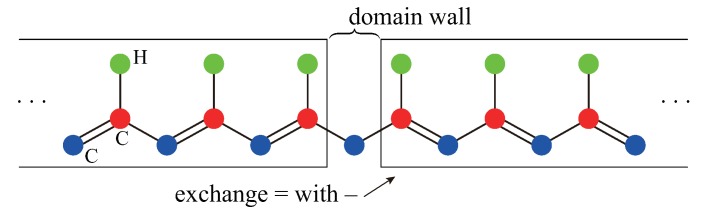
Two semi-infinite polyacetylene chains with a domain wall, which connects two different dimerized phases labeled −m and *m*. A double bond is shorter than a long bond and a Peierls lattice distortion is present. The directions of the double bonds vary in these two phases. Periodic boundary conditions cannot be imposed on this system as there is only one domain wall. Either a soliton or antisoliton is possible, but not both.

**Figure 3:**
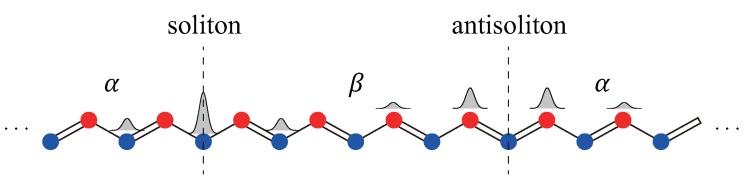
In a periodic system with two domain walls, a soliton-antisoliton pair must exist. Their site probability densities are shown. For a soliton (antisoliton), the probability density is finite on A (B) carbon atoms only.

Consider two semi-infinite polyacetylene specimens. A domain wall connects two degenerate ground states of the polyacetylene, as shown in Figure 2. In the continuum description of this system, a fermion mass potential in the Dirac equation produces an excitation gap, and a twist in this scalar potential produces a *zero*-energy soliton and fermion fractionalization [9]. The Dirac equation [21] has the form

(11) $$\begin{array}{c} H=-iv\hbar \partial_{x}\sigma_{x}+m\left(x\right)v^{2}\sigma_{y}, \end{array}$$

where $\left\{\sigma_{x},\sigma_{y}\right\}$ are Pauli spin matrices and *v* is the characteristic velocity. The second term is the twisted scalar mass potential. The twist is −m for x<0 and *m* for x>m, where *m* is a constant (this system is not periodic as the ±m terms represent two different dimerized phases). This equation has *one* zero-energy soliton (kink) mode $\psi_{0}\left(x\right)$, which is bound to the domain wall and decays exponentially. Its first and second components give the probability amplitudes of finding the electron on A and B carbons, respecitvely. However, only the A-component of the wavefunction is non-zero (this solution corresponds to a soliton that is very well localized near the A-carbon atom at x=0, as shown in Figure 3). A soliton *is a chiral mode* and is topologically robust, as it originates from a twist in the variation of the dimerization m(x).

The following discussion demonstrates that the conduction and valence bands each contribute a fractional spectral weight of 1/2 to a soliton. For simplicity, *spinless* electrons in disorder-free and half filled polyacetylene are considered first. The conduction and valence bands obey chiral symmetry so that, for opposite energies *E* and −E, there are identical wave functions: $\psi_{E}\left(x\right)=\psi_{-E}\left(x\right)$ (particle-hole symmetry follows from chiral symmetry; the Fermi energy is $E_{F}=0$). A spectral analysis is performed using the local density of states of the *translationally invariant* and non-invariant systems, $\rho^{0}\left(E,x\right)$ and $\rho^{kink}\left(E,x\right)$, respectively. As the total weight is conserved before and after the translational symmetry is broken, we have

(12) $$\begin{array}{c} \sum_{E}\rho^{kink}\left(E,x\right)=\sum_{E}\rho^{0}\left(E,x\right). \end{array}$$

Before a soliton is introduced there is no zero-energy state; thus, $\sum_{E}\rho^{0}\left(E,x\right)=\sum_{E\neq 0}\rho^{0}\left(E,x\right)$. After a kink is introduced, the total density of states (DOS) at *x* is $\sum_{E}\rho^{kink}\left(E,x\right)={\left|\psi_{0}\left(x\right)\right|}^{2}+\sum_{E\neq 0}\rho^{kink}\left(E,x\right)$. From this and the closure property of the eigenstates $\psi_{E}\left(x\right)$

(13) $$\begin{array}{c} \sum_{E}\rho \left(E,x\right)=\sum_{E}\psi_{E}^{+}\left(x\right)\psi_{E}\left(x\right)=1, \end{array}$$

we find

(14) $$\begin{array}{c} \sum_{E\neq 0}\rho^{0}\left(E,x\right)={\left|\psi_{0}\left(x\right)\right|}^{2}+\sum_{E\neq 0}\rho^{kink}\left(E,x\right). \end{array}$$

The induced DOS excluding the E=0 state is

(15) $$\begin{array}{c} \sum_{E\neq 0}\delta \rho \left(E,x\right)=\sum_{E\neq 0}\left(\rho^{kink}\left(E,x\right)-\rho^{0}\left(E,x\right)\right)=-{\left|\psi_{0}\left(x\right)\right|}^{2}. \end{array}$$

As the conduction and valence bands are symmetric, the contribution from the *occupied* valence band states is

(16) $$\begin{array}{c} \sum_{E<0}\delta \rho \left(E,x\right)=-\frac{1}{2}{\left|\psi_{0}\left(x\right)\right|}^{2}. \end{array}$$

Thus, the occupied valence band contributes a *fraction* of 1/2 to the total spectral contribution of the solitonic state, while the unoccupied conduction band contributes another half (one half of a state is missing from the valence band and the corresponding charge is assumed to be in the vicinity of the soliton [11]).

When the electron spin is considered, each spin-up and -down soliton gap state takes half the spectral weight from the valence band. Thus, if the spin-up and -down soliton states are both empty, the localized soliton has Q=e/2+e/2=e and S=0 [10,11] (here, a charge is defined as a depletion or surplus in the many-body *ground state density* including the positive background charge). When the spin-up soliton state is occupied while the spin-down state is empty, Q=−e+(e/2+e/2)=0 and S=1/2. Therefore, these solitonic states have unusual charge and spin relations [24].

Next, consider a *periodic* polyacetylene specimen having two domain walls, see Figure 3. It is instructive to consider tight-binding solutions. They are a soliton and an antisoliton solutions, as shown in Figure 3. For infinitely long polyacetylene, the energy difference between a soliton and antisoliton vanishes. When both a soliton and antisoliton are present, they must be located at *different* positions along a periodic ring: a soliton connects two dimerized phases, −m→m, whereas an antisoliton connects m→−m, as shown in Figure 3. A soliton and an antisoliton are chiral modes with different chirality: a soliton has only the A-component wavefunction and an antisoliton has only the B-component wavefunction, as apparent from Figure 1a and Figure 3. An antisoliton is also an eigenstate of the chiral operator. The energy spectrum varies strongly in the vicinity of the kink/antikink. The total number of electrons or states in the filled valence band in the vicinity of the kink/antikink decreases by precisely 1/2 per spin (see the derivation below).

### 3.2. End Solitons of Polyacetylene

**Figure 4:**
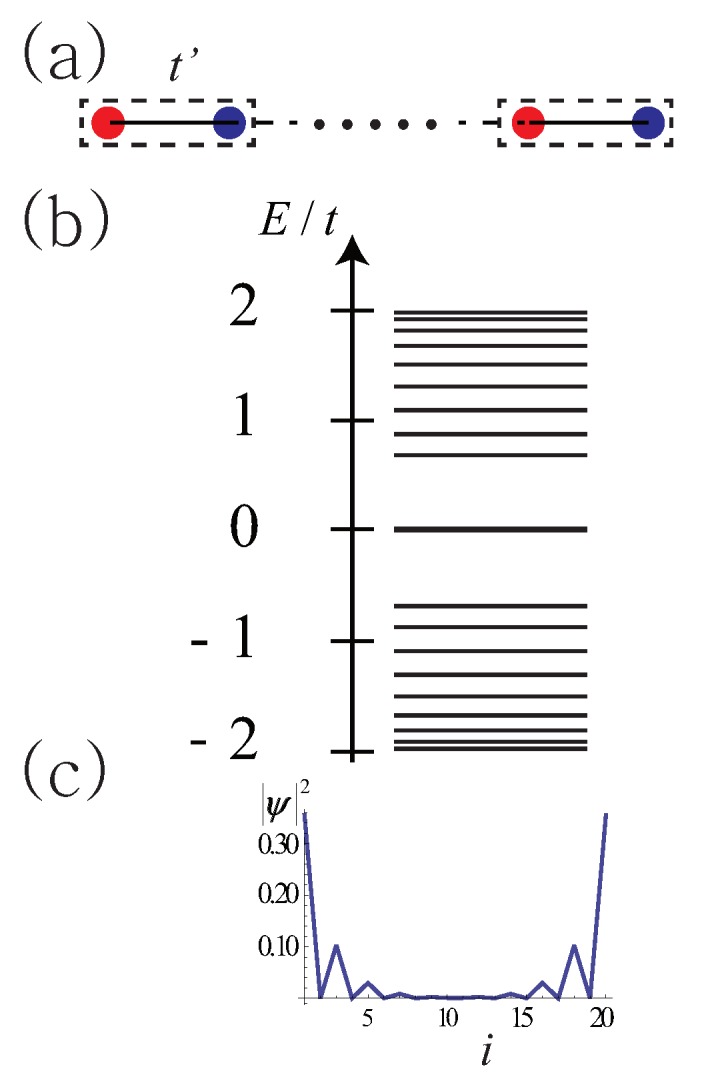
(**a**) Finite-length dimer chain with unit cell containing two carbon atoms connected by single bond. The Intra cell hopping $t^{\prime }$ is smaller than the inter cell hopping *t*. (**b**) Tight-binding energy spectrum. Two nearly degenerate gap states exist. (**c**) Probability density of gap state as function of site index *i*. A peak is apparent at the red (blue) site at the left (right) end. The probability densities of the bonding and antibonding states are almost identical.

**Figure 5:**
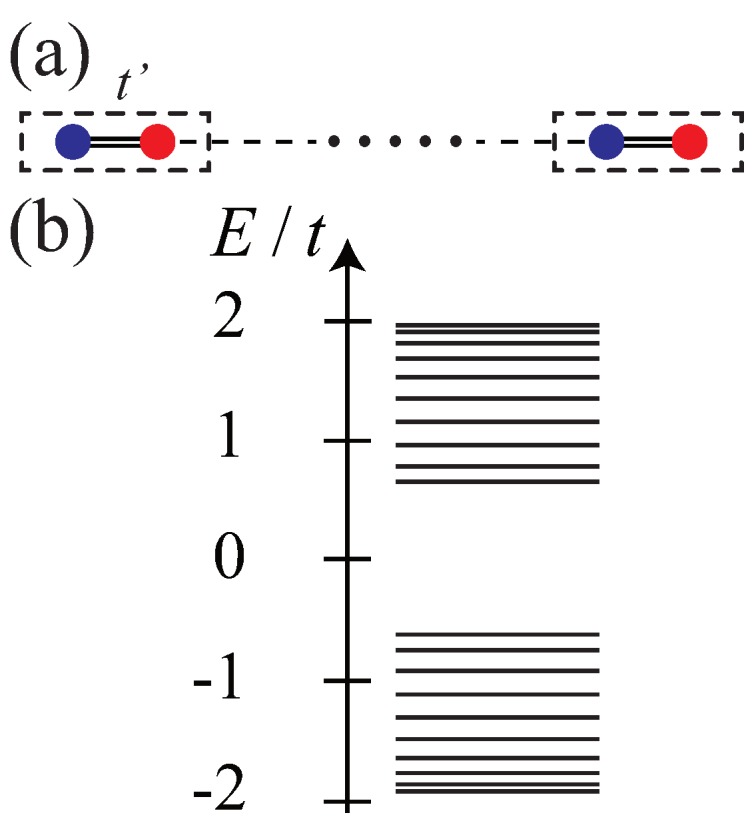
(**a**) Finite-length dimer chain with unit cell containing two carbon atoms connected by double bond. The intra cell hopping $t^{\prime }$ is larger than the inter cell hopping *t*. Note that the unit cell is different from that shown in Figure 4. (**b**) Energy spectrum. No gap states exist.

A soliton can also exist as a boundary charge. Consider *finite-length* polyacetylene in one of the dimerized phases (no domain wall exists as only one type of dimerized phase is present). The electron density is uniform with occupation number $n_{i}=1$ at all sites *i*. There are two types of *finite* length polyacetylene, which have long or short end bonds, as shown in Figure 4 and Figure 5, respectively.

Tight-binding calculations show that, for the long-bond unit cell shown in Figure 4a, two nearly degenerate bonding $\phi_{B}$ and antibonding $\phi_{A}$ *gap* states exist with almost zero-energy (the energy splitting vanishes when the system length becomes infinitely large). One half of the spectral weight of each of these solitonic states is derived from the conduction band, while the other half is from the valence band. As is $E_{F}=0$, one state is occupied while the other is empty (see Figure 4b). The probability density of such a state *splits* into two parts, located near the left and right end points (see Figure 4c). If an electron is added to a solitonic state, the resulting electron density ρ(x) has −e/2 fractional charges near the *two ends* of the polyacetylene. When an electron is removed, e/2 fractional charges appear near the ends (there is as yet no conclusive experimental evidence of their existence). Note that these solitons have mixed chirality with different chirality at the opposite ends (here referred to as *here refered to as mixed chiral states*). However, their linear combinations $\phi_{B}\pm \phi_{A}$ are *chiral* and are located near either the left or right end points. In the case of the short-bond unit cell, no end state exists, i.e., no gap state exists, as shown in Figure 5. In periodic polyacetylene, a topological phase transition occurs at $t=t^{\prime }$ with a discontinuous change in the value of the Zak phase (see the discussion of the Zak phase and end charge in subsection II C).

## 4. Solitons in Insulating GNR

According to tight-binding calculations [25,26], an AGNR has a gap and is semiconducting when the transverse width is $L_{y}=\left(3M+1\right)a_{0}$ or $3Ma_{0}$, where $a_{0}=\sqrt{3}a$ is the unit cell length of the graphene lattice, a=1.42 Å is the carbon-carbon distance, and *M* is an integer. However, when $L_{y}=\left(3M+2\right)a_{0}$, the AGNR has no gap and is metallic (we do not consider this case here). A rectangular GNR has two zigzag edges and two armchair edges. When the zigzag edges are longer than the armchair edges, a ZGNR is realized. In the opposite case, an AGNR is realized. A periodic ZGNR with a bandstructure has only two zigzag edges and no armchair edges.

Graphene lattice is two-dimensional, unlike that of polyacetylene. Chiral symmetry then leads to a phenomenon called “fermion doubling” [27] (K and K’ valleys exist). It implies that a GNR with the shortest width has *two* domain-wall states, in contrast to polyacetylene (for other values of the width the number of zero modes is an even integer [13]). It should be also noted that a soliton mode of a GNR that we describe below connects sites with different chiralities. It can connect two well-separated topological zigzag edges with opposite chiralities, and hence it is topologically protected. On the other hand, Sasaki et al. [12] explored a graphene nanoribbon with a domain-wall soliton connecting two distinct bonding structures (each structure has a bond alternation similar to dimerization in polyacetylene).

### 4.1. Domain-Wall Soliton

We describe below a domain-wall soliton of a semiconducting AGNR under a local tensile strain [13]. Such a domain-wall does not require Kekulé-like bond alternation. Some of the salient features of the domain-wall soliton are discussed in this subsection, and the similarities and differences in comparison to those of polyacetylene are delineated.

**Figure 6:**
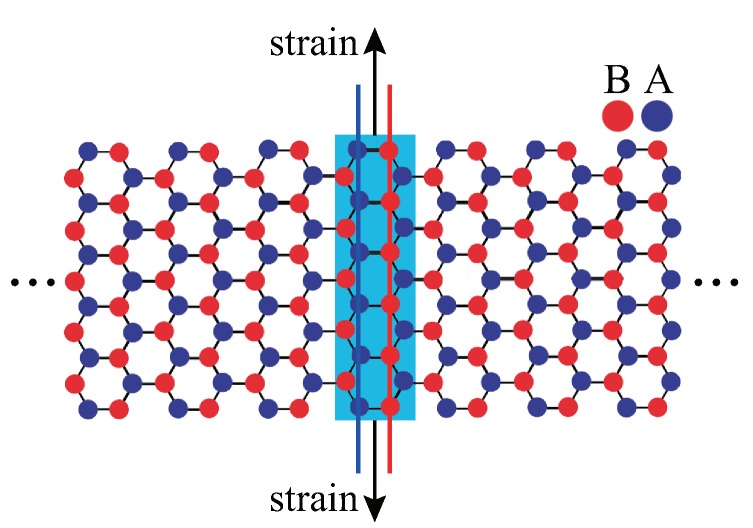
AGNR with local tensile strain applied perpendicular to ribbon direction in region D (blue area). A and B zigzag edge sites are shown on the blue and red lines.

Consider an infinitely long semiconducting AGNR. Suppose we apply a local tensile strain perpendicular to the ribbon direction [13], as shown in Figure 6. This induces changes in the hopping parameters between the carbon atoms in a rectangular area *D*, where strain is applied. In the continuum description of graphene, such a distortion can be simulated by a chiral gauge field ${\vec{A}}_{f}\left(\vec{r}\right)$ [12]. For the *K* valley, we have

(17) $$\begin{array}{c} H_{K}=v_{F}\vec{\sigma }\cdot \left(\vec{p}-\frac{e}{c}{\vec{A}}_{f}\left(\vec{r}\right)\right), \end{array}$$

where the Pauli spin matrices are $\vec{\sigma }=\left(\sigma_{x},\sigma_{y}\right)$, $\vec{p}$ is the momentum operator, and $v_{F}$ is the Fermi velocity of bulk graphene. The second term is the chiral gauge vector field. Similarly, for the $K^{\prime }$ valley, we have

(18) $$\begin{array}{c} H_{K^{\prime }}=v_{F}{\vec{\sigma }}^{\prime }\cdot \left(\vec{p}+\frac{e}{c}{\vec{A}}_{f}\left(\vec{r}\right)\right), \end{array}$$

where ${\vec{\sigma }}^{\prime }=\left(-\sigma_{x},\sigma_{y}\right)$. Note that the signs of the chiral gauge vector field differ in these equations. The chiral vector is a constant in *D*, its direction is along the x-axis, and it is zero outside *D*. However, it is not a real vector potential; rather, it effectively describes the change in the hopping parameters in *D*, i.e., in the domain wall. The armchair edges *couple* the two valleys and the solutions are four-component wave functions [25].

**Figure 7:**
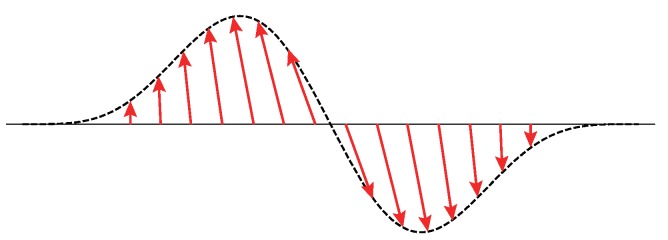
Domain-wall soliton corresponds to a topological kink. Pseudospin vector rotates by an angle of π out of the plane.

For a ribbon with the shortest width these equations have *two* solutions with E≈0, representing gap states, i.e., a domain wall supports *both* solitonic and antisolitonic solutions with opposite energies [13]. The antisoliton state is unoccupied at half filling. *Half* the spectral weight of a soliton/antisoliton arises from the conduction band and *the other half* from the valence band. The solitonic states are *not* eigenstates of the chiral operator: on the left and right hand sides of the domain, the chirality of the dominant wavefunction is of A- and B-type, respectively (see Figure 6). These states are mixed chiral states (see Figure 1d). However, a soliton and antisoliton are connected to each other by chiral operation (they have opposite energy). When the tensile strain is sufficiently strong that the bonds in region *D* almost break, the left and right parts of the wavefunction have a rather small overlap. Note that the pseudospin [28] of the solitonic state rotates by π across the domain wall, indicative of a topological kink [29], as shown in Figure 7. The crucial feature of a topological kink is that the total change is π irrespective of the manner in which the phase changes as the coordinate changes. Unusual spin and charge relations are not expected in GNRs [20], as a *single* domain wall can simultaneously support both a soliton and an antisoliton, in contrast to polyacetylene. Note that, when both the soliton and antisoliton states are unoccupied, the valence band misses the total charge of *e* per spin.

**Figure 8:**
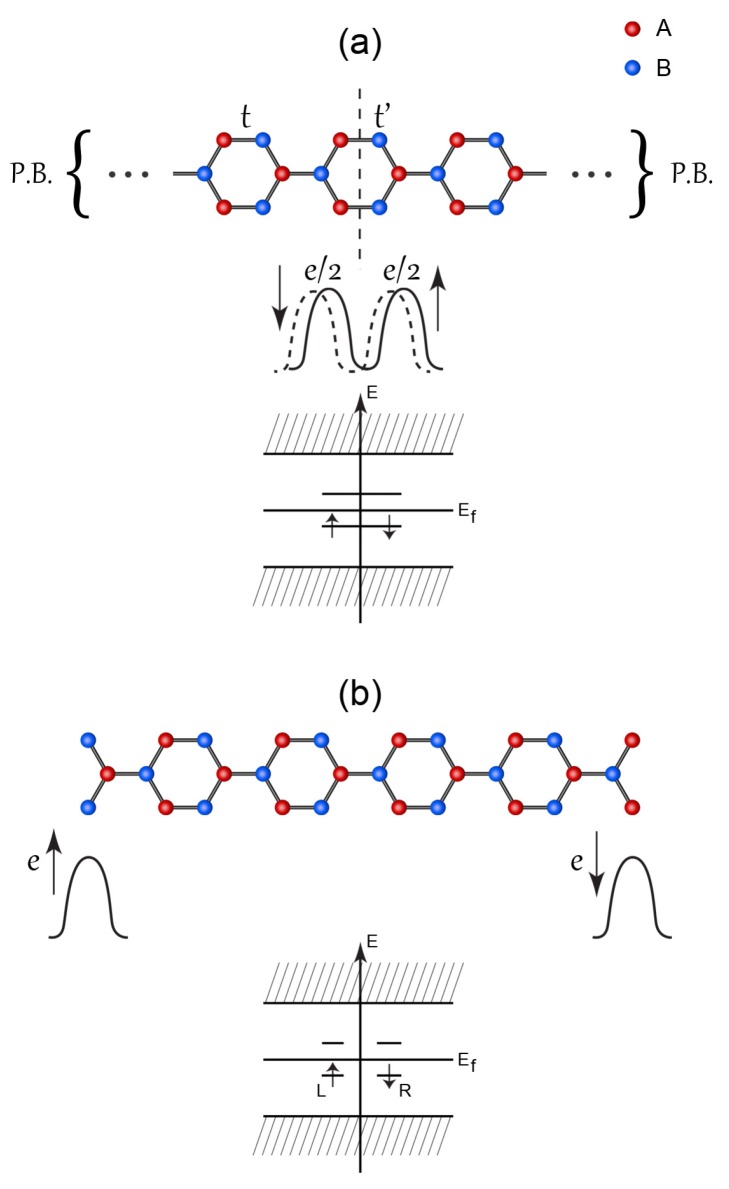
(**a**) Periodic armchair graphene nanoribbon (AGNR) with shortest width and modified hopping $t^{\prime }$ between two neighboring zigzag edges at center. P.B. represent periodic boundary conditions. A schematic illustration of the probability densities of the soliton gap states is also shown. Some overlap between its left and right probability densities occurs if the hopping between these two edges, i.e., $t^{\prime }$, is non-zero. The energy spectrum of the gap states for a finite on-site electron repulsion *U* is also shown. In the limit of small *U*, the energy splitting of the solitonic gap states vanishes and the energies approach zero. (**b**) In the limit $t^{\prime }\to 0$ (corresponding to cutting of the bond), a rectangular AGNR is realized. The solutions change qualitatively: antiferromagnetically coupled integer charges develop that are localized on the left (L) and right (R) zigzag edges.

The domain-wall soliton may be constructed from a linear combination of chiral zigzag edges modes. It is instructive to analyze this problem using a simple model [13]. Suppose we consider a periodic AGNR with a *short* width, and assume, for simplicity, that only one bond is affected by tensile strain, as shown in Figure 8a. When the distorted hopping parameter vanishes, $t^{\prime }=0$, the bulk-edge correspondence indicates that *chiral* modes $\phi_{L}$ and $\phi_{R}$ develop on the left and right zigzag edges (see Figure 1c). When $t^{\prime }\neq 0$, a domain soliton mode forms, which can be expressed as a linear combination $\phi_{L}\pm \phi_{R}$ of the chiral modes of the left and right zigzag edges at $t^{\prime }=0$, as shown in Figure 8a (both bonding and antibonding combinations are possible). An electron in a solitonic state resides near the two neighboring zigzag edges with *opposite chirality* [13]. Its tight-binding probability density is divided equally between the left and right zigzag edges (the solitonic wavefunction exhibits some overlap between the edges). This domain soliton mode is robust as the edge chiral modes $\phi_{L}$ and $\phi_{R}$ are topologically protected [4,22] (they persist as long as the zigzag edges are not destroyed). In the limit where $t^{\prime }\to 0$, a rectangular AGNR is realized, as shown in Figure 8b. In addition, the effect of on-site repulsion is more significant in comparison to its effect for $t^{\prime }\neq 0$. It makes the bonding and antibonding states $\phi_{L}\pm \phi_{R}$ no longer stable on the zigzag edges. This is because on-site repulsion induces antiferromagnetic coupling between edge charges and mitigates the formation of fractional edge charges.

### 4.2. Solitons of Interacting ZGNR

**Figure 9:**
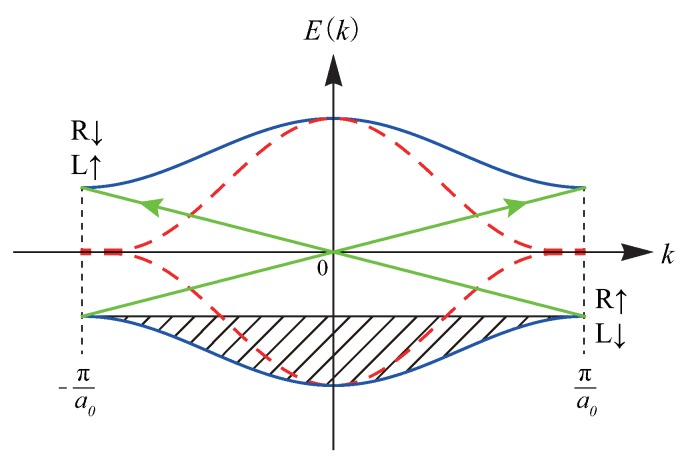
Schematic display of band structure for interacting ZGNR in absence of disorder (solid lines). (The gap size is exaggerated). Only bands near the gap are displayed. The natures of the unoccupied and occupied states near $k=\pm \frac{\pi }{a_{0}}$ are given ($a_{0}$ is the unit cell length of the ZGNR): *R* and *L* represent states localized on the right and left zigzag edges, respectively. Small arrows indicate spins. The bandstructure for non-interacting electrons is represented by dashed lines. There are numerous zero-energy states near the Brillouin zone boundary, which split in the presence of on-site electron repulsion *U* (this splitting is analogous to the energy splitting shown in Figure 8. The long arrow indicates coupling between states R↑ and L↑ or R↓ and L↓.

In this subsection, end solitons of half-filled periodic ZGNRs are considered. The Hartree-Fock approximation [15,30,31,32] (HFA) result for such a disorder-free system displays numerous pairs of occupied spin-up and -down chiral edge states that are located on the opposite zigzag edges. They are solitons of the type shown in Figure 1c [3]. These states correspond to states near the first Brillouin zone boundary, as shown in Figure 9. Their number is even and increases linearly with the zigzag edge length [13]. Electron interaction is responsible for the excitation gap and edge antiferromagnetism [3,33,34]. The study of the Zak phase also suggests that the edge charge on a zigzag edge is an integer [20]. There are no well-separated solitonic boundary charges of e/2 on the zigzag edges, as the antiferromagnetic coupling between the zigzag edges produces integer charges.

When a disorder-free ZGNR is doped, the additional electrons form a solitonic phase [15] (then, $E_{F}>0$). On the left zigzag edge, the edge spin profile exhibits a rotation of π as the coordinate position varies from one end of the zigzag edge to the opposite end of the same zigzag edge, i.e., it rotates from spin-up to spin-down. On the right zigzag edge, the edge spin profile rotates from spin-down to spin-up. The DOS develops a sharp solitonic peak at E=0 in the middle of the gap.

## 5. Soliton Fractional Charge of Interacting Disordered ZGNR

The domain-wall soliton of Figure 1d has two fractional charges that overlap. This is not a true charge fractionalization as the overlap between fractional charges is not small. As demonstrated in the previous section, neither of the two zigzag edges of a rectangular AGNR supports a fractional charge because of the edge antiferromagnetism, as shown in Figure 8b.

An impurity potential or magnetic field can have a significant probability density redistribution effect over the opposite zigzag edges [35,36]. For example, when the reflection symmetry of a rectangular AGNR is broken by a staggered potential, the Zak phase is no longer quantized and the zigzag edge charge can take non-integer values [20]. In this case the Zak phase can be related only approximately to the edge charge via Equation (3). Moreover, for some values of the strength of the staggered potential, numerous states of a rectangular AGNR are spin-split while the states of the corresponding periodic AGNR are not. For these GNRs the Zak phase cannot be related to the edge charge.

**Figure 10:**
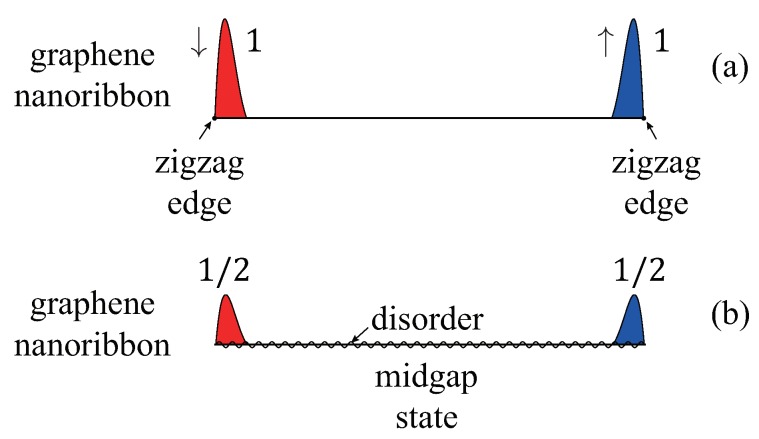
(**a**) In a clean and gapful ZGNR, the zigzag edges couple antiferromagnetically and no fractional boundary charge exists on them. An edge state of this system displays an integer boundary charge of one. The shaded areas in the figure indicate the probability densities of the edge states. Note that the chirality differs among the zigzag edges. (**b**) In a disordered ZGNR the antiferromagnetic coupling is partly weakened and a midgap state (E≈0) with a given spin value can display fractional end charges of e/2. Note that this state is localized along the zigzag edges, in contrast to the AGNR case shown in Figure 8b. The wavy line indicates a disorder potential.

Let us examine the effect of disorder on charge fractionalization in undoped interacting disordered ZGNRs. *The gap of a disordered ZGNR is filled with localized states, whereas the states outside the gap are delocalized* [16]. (The usual one-dimensional localization theory does not apply to GNRs [37,38]). A short-ranged disorder potential induces stronger localization along the zigzag edges than a long-ranged disorder potential [39]. In addition, spin-split states are also present [20,34], as in a Mott-Anderson insulator [40,41]. Let us analyze the scattering of the left and right edge states by a short-ranged disorder potential. Consider a spin-up electron at $k=\frac{\pi }{a_{0}}$ with the wavefunction $\phi_{R↑}$ localized on the right zigzag edge. For a short-ranged potential, a significant wave vector transfer in a backscattering occurs for $|k-k^{\prime }|∼1/a_{0}$ [39]. Such a short-ranged disorder potential couples the *chiral* zigzag edge state $\phi_{R↑}$ to another *chiral* zigzag edge state $\phi_{L↑}$ on the opposite zigzag edge at $k=-\frac{\pi }{a_{0}}$, as shown in Figure 9 ($\phi_{R}$ and $\phi_{L}$ are depicted in Figure 10a). This process produces the bonding $\frac{1}{\sqrt{2}}\left(\phi_{L}+\phi_{R}\right)$ or antibonding $\frac{1}{\sqrt{2}}\left(\phi_{L}-\phi_{R}\right)$ edge state. These states display charge fractionalization with 1/2 charges on the left and right zigzag edges. The probability density of one of these states is shown schematically in Figure 10b (a mixed chiral state). Moreover, when the disorder is weak, the DOS near the gap edges is *sharply peaked*. Because of this sharp peak, even a weak disorder potential can mix the left and right zigzag edge states and may generate edge states that are fractionalized between the opposite zigzag edges. These results suggest that solitonic fractional charges may exist in disordered ZGNRs.

The midgap states with E≈0 are of special interest. Note that the presence of the midgap states changes the magnetic properties of ZGNRs: the edge antiferromagnetism is weakened with the proliferation of midgap states. A self-consistent treatment of disorder and electron interaction within the HFA [42,43] shows that a midgap state of a given spin value can fractionalize into two e/2 fractional boundary charges on the opposite zigzag edges (see Figure 1e and Figure 10b). This state is divided into two equal parts and decays exponentially from the zigzag edges [38]. If an electron is added to a midgap state, the resulting total electron density ρ(x,y) has fractional charges on the zigzag edges. The wider the distance between the opposite zigzag edges, the better the fractional quantization, as the overlap between the fractional charges on the left and right zigzag edges decreases. This state represents a pseudospin kink connecting the left and right zigzag edges of different chiralities. Numerical calculations show that the charge fluctuations induced by disorder around the mean value e/2 are small in the weak-disorder regime [16]. However, these fluctuations increase as the strength and range of the disorder potential increase. To constitute true charge fractionalization, the quantum charge fluctuations should occur at high frequencies. According to Girvin [44], the characteristic time scale for the charge fluctuations is inversely proportional to the relevant excitation gap. There is a small gap between the occupied and unoccupied midgap states in the weak-disorder regime, and this gap induces very small time scales for quantum fluctuations [16]. It is possible for charge fractionalization to also occur on the zigzag edges of a long disordered AGNR (Figure 8b).

Other gap states in the energy interval [E−δE,E+δE] with E≠0 also have an average fractional boundary charge value of e/2: half the states in this interval are more localized on the left zigzag edge, with the other half being more localized on the right zigzag edge. Thus, their average boundary charge on one zigzag edge is e/2. However, they have larger charge fluctuations induced by disorder in comparison to the midgap states.

## 6. Summary and Conclusions

Polyacetylene and GRNs exhibit bulk-edge correspondence between the Zak phase and the existence of chiral boundary states; this is guaranteed by their chiral symmetry. In this paper, various chiral and mixed-chiral edge modes of polyacetylene and GNRs have been described. Mixed chiral states, formed by bonding and antibonding linear combinations of the chiral edge modes, play an important role in charge fractionalization. Weak disorder stabilizes soliton states with fractional edge charges in ZGNRs, in contrast to what is usually expected.

Polyacetylene and GNRs also have several different topological properties. In polyacetylene, a domain-wall soliton has unusual spin and charge relations. Moreover, when the intra cell hopping parameter is smaller than the inter cell hopping parameter, the polyacetylene is topologically non-trivial and fractional boundary charges exist with a charge of e/2. In the opposite scenario, however, the polyacetylene is topologically trivial with zero boundary charge. In other words, long-bond polyacetylene is topologically non-trivial, but short-bond polyacetylene is not. This reflects the fact that the bulk polarization depends on the choice of unit cell [18]. A topological phase transition exists when the intra and inter cell tunneling coefficients are equal.

Domain-wall solitons can also exist in GNRs. Unusual spin and charge relations are not present in the GNRs as a domain wall supports an even number of solitons (manifestation of “fermion doubling”); this is in contrast to the case of polyacetylene. Moreover, fractional boundary charges do not exist in disorder-free rectangular AGNRs and ZGNRs because of the antiferromagnetic coupling between the well-separated zigzag edges; i.e., only integer boundary charges can exist on the zigzag edges. The choice of unit cell is immaterial for GNRs, in contrast to polyacetylene. Disorder has profound effects on the zigzag edge states of GNRs. In the presence of disorder, a half-filled ZGNR becomes a Mott-Anderson insulator with numerous spin-split states. Moreover, the gap states are localized whereas the states outside the gap are delocalized. Additionally, a non-trivial interplay between disorder and electron interactions induces formation of a fractional boundary charge. A disorder potential, especially a short-ranged potential, is effective in partly mitigating the effect of antiferromagnetic coupling and can induce formation of a fractional charge of midgap states. Other gap states in a small energy interval also have e/2 average fractional charge, but with larger charge fluctuations. These gap-edge states represent topological kinks. Disorder thus changes topological properties of ZGNRs. Note that, in the case of ZGNRs, boundary charges exist on the side edges of the ribbon (one at each side).

A fractional boundary charge may be observed by adding or removing an electron from the midgap states. Scanning tunneling microscopy [45] may provide rich information on the values and fluctuations of the boundary charges of localized gap states. It may be worthwhile to develope a field theoretical description of charge fractionalization in interacting disordered ZGNRs. A particle with a fractional charge is usually an anyon [46]. More theoretical work is needed to establish this aspect, as detection of a possible anyon state in GNRs would be most interesting.
